# An economic evaluation of an intervention to increase demand for medical male circumcision among men aged 25–49 years in South Africa

**DOI:** 10.1186/s12913-021-06793-7

**Published:** 2021-10-15

**Authors:** M. Holmes, R. Mukora, D. Mudzengi, S. Charalambous, C. M. Chetty-Makkan, H. Kisbey-Green, M. Maraisane, J. Grund

**Affiliations:** 1 Centers for Disease Control and Prevention, Atlanta, GA USA; 2 Economics Department, Spelman College, 350 Spelman Lane, Atlanta, GA 30314 USA; 3 The Aurum Institute, Johannesburg, South Africa; 4 The School of Public Health, University of the Witwatersrand, Johannesburg, South Africa; 5 Health Economics and Epidemiology Research Office, Johannesburg, South Africa; 6 Centers for Disease Control and Prevention, Pretoria, South Africa

**Keywords:** Demand creation, Medical male circumcision, HIV, Cost-effectiveness analysis, Cost-benefit analysis

## Abstract

**Background:**

Studies estimate that circumcising men between the ages of 20–30 years who have exhibited previous risky sexual behaviour could reduce overall HIV prevalence. Demand creation strategies for medical male circumcision (MMC) targeting men in this age group may significantly impact these prevalence rates.

**Objectives:**

The objective of this study is to evaluate the cost-effectiveness and cost-benefit of an implementation science, pre-post study designed to increase the uptake of male circumcision for ages 25–49 at a fixed MMC clinic located in Gauteng Province, South Africa.

**Methods:**

A health care provider perspective was utilised to collect all costs. Costs were compared between the standard care scenario of routine outreach strategies and a full intervention strategy. Cost-effectiveness was measured as *cost per mature man enrolled* and *cost per mature man circumcised*. A cost-benefit analysis was employed by using the Bernoulli model to estimate the cases of HIV averted due to medical male circumcision (MMC), and subsequently translated to averted medical costs.

**Results:**

In the 2015 intervention, the cost of the intervention was $9445 for 722 men. The total HIV treatment costs averted due to the intervention were $542,491 from a public care model and $378,073 from a private care model. The benefit-cost ratio was 57.44 for the public care model and 40.03 for the private care model. The net savings of the intervention were $533,046 or $368,628 - depending on treatment in a public or private setting.

**Conclusions:**

The intervention was cost-effective compared to similar MMC demand interventions and led to statistically significant cost savings per individual enrolled.

**Supplementary Information:**

The online version contains supplementary material available at 10.1186/s12913-021-06793-7.

## Background

Medical male circumcision (MMC) has been confirmed to reduce the risk of HIV infection in men through heterosexual sex by at least 60% [1, 2]. The World Health Organization (WHO) and the Joint United Nations Programme on HIV/AIDS (UNAIDS) recommended that MMC be scaled up in priority countries with high HIV prevalence and low male circumcision coverage [3]. As of 2018, South Africa had an HIV prevalence of 20.4% among adults aged 15–49 years [4]. A mathematical modelling study showed that circumcising men between ages 20–30 years with risky sexual behaviour could reduce overall HIV prevalence [5]. Other societal health benefits of MMC are the number of HIV cases that could be averted following the MMC procedure [6–8] and averted HIV care costs among those circumcised and their sexual partners [9, 10].

As of 2016, over 2.84 million South African adolescent and adult males were circumcised as part of the South African MMC for HIV prevention program [11], yet MMC coverage varied substantially by province [12]. Despite circumcising a large number of males, the proportion of circumcised males aged 15–49 years is reported to be only 46% [5]. Among South African men, only 37% of men aged 25–49 years and 12% of men aged ≥50 years indicated interest in becoming circumcised [13]. Circumcising males aged 20–34 years could provide the most immediate effect on HIV incidence, and one modelling study in South Africa identified the most cost-effective age group for MMC was men aged 15–29 years [5].

With the focus on having the most immediate and cost-effective impact on HIV incidences, a MMC scale-up strategy for South Africa should include men aged ≥20 years, yet this age group has been the most challenging for MMC demand creation. Demand creation refers to activities to promote MMC and identify potential eligible clients. Data for this economic evaluation were collected from an implementation science, pre-post study (named “Imbizo”) that evaluated an intervention to increase the uptake of MMC among men aged 25–49 years at a fixed MMC clinic in Gauteng Province, South Africa. Main findings from this implementation science study have been presented elsewhere [14].

## Methods

### Description of the study

The “Imbizo” study was nested within a routine MMC service delivery program that consisted of two phases: Phase 1 (referred to as the “standard care”) took place from 1 April – 30 September 2014 and Phase 2 (referred to the “intervention”) occurred from 22 June – 30 November 2015. More detailed study procedures have been previously published elsewhere [14]. In summary, data were collected in Phase 1 on the baseline risk factors for men presenting for MMC aged 18–49 years. Qualitative data on barriers and motivators for MMC were also collected during this phase. Interventions were developed based on the themes from the qualitative data, which were implemented in Phase 2. For example, prior programmatic experience and previous studies on MMC demand creation show that adult men prefer to sit in separate waiting areas for MMC surgery - away from younger adolescent boys, as their mothers usually accompany the boys for parental consent purposes [15]. For this reason, one of the components of the Phase 2 “Exclusive Intervention Strategy” was to divide the facility into two parts where adult men would be in a physically separate area from younger clients.

The “Exclusive Intervention Strategy” included a combined approach of infrastructure changes at the MMC facility to separate adults (aged ≥18 years) from adolescents (aged 10–17 years). Other components included a men’s health club, which contained a lounge area, free Wi-Fi access, VIP Facebook page by invite only, and shoeshine services, all for men aged ≥18 years. Also, only available at the adult study clinic, were adult-specific community demand generation materials – specifically billboards, pamphlets, posters and branded condoms. Another component, “Imbizo” (traditional meetings where elders discuss important issues) discussions were held with community members. The “Imbizo” concept for this study (also called the “community intervention”) included broader community discussions with both men and women regarding MMC [14]. The “Exclusive Intervention Strategy” in Phase 2 was aimed at increasing the number and proportion of men aged 25–49 years present for MMC. This “Exclusive Intervention Strategy” further took place at the clinic and in the community and included the five aforementioned strategies that are described in Table 1.

**Table 1:** Activities for the Exclusive Intervention Strategy that targeted uncircumcised adult men in Gauteng province, South Africa, 2015

	Type of activity	Description of activity
1	Structural modifications at the circumcision clinic	A separate space was created for adult men (ages ≥18 years) to provide privacy and separation from adolescents (10–17 years old)
2	VIP Facebook Club	VIP Facebook page by invite only: Exclusive group access to a Facebook page for those between 25 and 49 years. Those in the club received information on men’s health from a clinician and had a platform to ask questions that they found difficult to pose in a group.
3	“Imbizo” Meetings (or the community intervention)	“Imbizo” is a Zulu word meaning a forum where elders of the community meet to address issues concerning individuals, family and other community matters. This was a discussion forum that men attended before they were invited to be circumcised. During this discussion, information on the MMC procedure, six-week sexual abstinence, healing time after circumcision, myths on MMC, what happens to the foreskin after MMC and general health issues was addressed. If men indicated that they also wanted their partners to join the discussion, this was arranged to accommodate their needs.
4	Male friendly services	Lounge waiting area Free Wi-Fi access Shoeshine services
5	Community demand generation material	Outreach branded messages, materials and male adverts that were tailored specifically to address the issues of men (aged 25–49 years) and MMC. These included billboards, pamphlets, posters and branded condoms

### Methodological approach

This study evaluated the cost-effectiveness and cost benefit of the Imbizo program. A health care providers’ perspective (which includes those who provided and paid for treatment) was employed to examine the costs and consequences of the intervention. Costs were evaluated for 13 months, which was over the timeframe for both phases of the Imbizo study. Costs were collected using a bottom-up approach and only financial costs (direct medical costs) were included. Patient costs were excluded, as it was assumed that participants would not have incurred any additional costs for MMC. Standard care costs for recruitment and routine MMC service delivery were collected retrospectively and intervention costs for the “Exclusive Intervention Strategy” were collected prospectively. The remaining cost categories included: personnel, supplies, training, travel, utilities, equipment, vehicles, contracted services, structural modifications and buildings.

Though the “Exclusive Intervention Strategy” was delivered as part of a package, costs for the intervention were allocated to each component of the intervention. Costs were grouped as recurrent or capital. Recurrent costs were those which had a useful life of less than a year or had a purchase price of ≤100 United States dollars (USD). Capital costs were those with a useful life of more than a year and a purchase price of > 100 USD. Capital costs were amortized at 3%, and over a period of time designated by established accounting principles guidelines in South Africa [16]. All costs were captured in South African Rand (ZAR) and converted to the USD using the 2014 average exchange rate (1 USD = 13.88 ZAR). Structured interviews were conducted with the provider’s key personnel from finance, administration, and implementation departments (HIV testing services (HTS) & MMC). We excluded costs of research, pre-surgical MMC counselling and the circumcision surgical procedure, as these costs were incremental costs that did not influence the decision to use these activities. During the implementation science study, men aged 25–49 years old were recruited for the study. Among those men recruited, participants were subsequently enrolled. The number of men enrolled in the standard care were 877 and 722 in the intervention. (Enrolled refers to eligible men that passed the screening and were included in the main implementation science study.) A consort diagram of the enrolled sample are described in detail and published elsewhere [14].

## Recurrent costs

### Personnel

Total personnel costs included salaries of direct provision staff who worked full-time on HTS and MMC, along with managerial staff. HTS staff referred eligible participants for MMC and worked with the MMC team on demand creation campaigns. Using available standard care data, HTS and MMC staff were assigned a unique identifier to clearly identify each staff person’s recruitment numbers, activities, and time spent on MMC demand creation. A trained research assistant conducted interviews with the HTS and MMC staff to verify the completed activities. Managerial costs included staff members who worked indirectly with the intervention - specifically the data manager, project manager, and program director. Implementing partner leadership was interviewed, where it was determined that managers’ time were allocated as 50% on MMC activities and 50% on HTS. For the intervention scenario, personnel included staff working on MMC, HTS, implementing the “Exclusive Intervention Strategy” activities, and managers. Managers completed study-specific monthly timesheets while all other personnel completed daily timesheets for purposes of tracking study-based activities. All timesheets were saved on an internal, electronic system with restricted access.

### Supplies and utilities

For both the standard care and the intervention, invoices, purchase orders, and financial records were used to obtain the prices and quantities of supplies and utilities consumed. Supplies included items distributed during demand creation outreach activities such as stationery, pamphlets, posters, lanyards, and woollen hats. Unit costs were multiplied for materials as indicated on the invoices with the quantity consumed and summed over all materials to obtain total costs. Utility costs included water, telephone, electricity, and internet. The municipality bills for the circumcision facility to estimate electricity, water, and ablution costs were not obtained, therefore the costs per square metre of the municipality bills charged for the organization’s head office to the MMC facility was applied.

### Contracted services

Contracted services included bulk text messages (SMS) for demand creation and refreshments for the Imbizo discussions. Amounts were obtained from financial records.

### Building rental

As the MMC clinic building costs were not available, the rental rate for a nearby clinical research centre with similar measurements was used. To calculate the rental cost for both phases, the months of duration for the standard care and intervention were multiplied with the rate per square metre, and allocated costs to each phase accordingly.

## Capital costs

### Equipment and vehicles

Equipment included laptops, tables, plastic chairs, and vehicles. The full replacement costs of each equipment from public retail stores were obtained and annuitized with a 3% discount rate and the useful life of each equipment. The useful lives included 2 years for plastic chairs, 5 years for wooden furniture, and 10 years for laptops. The equivalent monthly cost of each item over the 6 months when the intervention was implemented was summed to obtain total cost of equipment.

There were three vehicles used during the standard care phase one of which was purchased for routine MMC activities during the intervention. To determine the cost of the vehicles used during the standard care, the travel logs indicating the date the vehicle was used were obtained and captured, followed by the purpose of the trip (i.e. if the vehicle was used for an MMC or HTS activity), and the odometer readings. The mileage for the standard care phase was separated from that of the intervention. The distance travelled for MMC activities during the intervention was divided by the total distance travelled to obtain the percentage of the costs to be allocated to MMC. Vehicle costs included the purchase price of the vehicle with a useful life of 10 years and a 3% discount rate.

### Training

Since no HTS trainings were conducted during the standard care, records from the last HTS training (2011–2012) were used. For the intervention, research assistants were trained on how to conduct Imbizo meetings. Training included standard operating procedures and how to use an interview guide to probe participants during the discussion. Training costs included venue, accommodation, supplies, projectors, and refreshments. The trainer’s time was accounted for within our personnel costs. All training costs were considered as human capital costs. These costs were therefore amortized over 3 years at 3%.

### Structural modifications

Financial records were used to obtain the construction costs to separate the adult waiting room from the adolescents. The costs were annuitized at 3% over 10 years and halved to obtain the six-month equivalent (the length of the intervention period) cost.

### Estimating cases of HIV averted and costs averted

Circumcision reduces the transmission of HIV. We therefore used a mathematical model to estimate the number of HIV cases averted due to circumcision. For the intervention phase, the Bernoulli model of STD infections was used to estimate primary (participants) and secondary (sex partners of participants) HIV infections averted. This model translated the number of sexual partners, the number of sex acts per partner, the use of condoms, and the effect of circumcision on HIV transmission into averted cases of HIV. (Refer to the supplemental material for detailed information on the model.) The Bernoulli model also considers the influence of key biological and epidemiological parameters on the probability of HIV infectivity. The intervention offered the benefit of averting HIV cases and associated medical costs (i.e. treatment, diagnostic testing, and clinician costs). The base-case value of the direct cost of HIV was based on published studies of the annual and lifetime antiretroviral therapy (ART) costs per person [17, 18].

### Cost-benefit measures

The net benefit and the cost-benefit ratio (CBR) were calculated. The net benefit (cost) was calculated as Net Benefit (cost) = (PV benefit – PV cost), where  is the present value of averted medical costs, and  is the present value of costs associated with implementing the intervention. Net benefit (cost) is the cost savings (or costs) that an intervention generates to society. The Cost-Benefit Ratio (CBR) was calculated as BCR = PV Benefits / PV Costs. Interventions are considered cost beneficial to society when the net benefit > 0 and the CBR is greater than 1. Averted costs for the Cost-Benefit Ratio examined published cost estimates of the private-care and public care models of providing care to public sector dependent patients [18].

### Sensitivity analysis

After calculating the base-case results, a one-way and multivariate sensitivity analyses was conducted using @RISK by Palisades to examine how the base-case results would change over a range of epidemiological values for each important model input. One parameter was varied at a time while holding all other parameters at their base-case values. All parameters were allowed (except female to male transmission rate) to vary for our multivariate analysis.

## Results

### Costs of the standard care and intervention

The total number of men (aged 25–49 years) included for the analysis from the standard care and intervention were 877 and 722, respectively. Table 2 shows the general cost categories and input for the base-case and the intervention. The total standard care cost for recruitment and subsequent enrolment was $15,780 for 877 men and $9445 for 722 men in the intervention in 2014/2015. This translated into unit costs of $18 and $13 for the standard care and intervention, respectively. The incremental cost of moving from the standard care to the intervention strategy was a cost-savings of $6336. For recurrent costs, approximately 83.9% was for the standard care and 78% intervention. Capital costs were 16% in standard care and 21% in the intervention. The major cost drivers for the standard care and intervention were personnel (64 and 52% of total cost, respectively), supplies (16 and 21%, respectively), and equipment (12 and 11%, respectively). Table 3 presents the costs for each strategy within the intervention. The five separate intervention strategies ranged from $42 to $$9445. The cost to construct a wall to provide men aged ≥18 years were low; while the cost to implement community intervention was significantly higher at 174 times the cost of the dry wall. Community intervention costs included personnel costs for 21 MMC recruiters, a program manager, and a counsellor. Salary costs were added for the oversight of intervention (i.e. salary processing etc). There were also substantial costs for promotional poster displays on commuter trains. The VIP Facebook page, male friendly services, and Imbizo meetings were lower cost interventions. Specifically, demand creation was 78% of costs, while the VIP Facebook page and Imbizo meetings were 11 and 6% of total costs, respectively.

**Table 2:** Costs for men aged 25–49 years receiving MMC in the standard care and intervention strategy in Gauteng Province, South Africa 2015 by input

Input	Standard care			Intervention		
	Total cost men aged 25–49 years (USD)	Unit cost per man aged 25–49 years receiving MMC (USD)	%	Total cost men aged 25–49 years (USD)	Unit cost per man aged 25–49 years receiving MMC (USD)	%
**CAPITAL COSTS**						
Equipment	1961.94	2.24	12.4%	1029.71	1.43	10.9%
Buildings	232.55	0.27	1.5%	458.10	0.63	4.8%
Training	303.46	0.35	1.9%	365.79	0.51	3.8%
Vehicles	33.64	0.04	0.3%	107.72	0.15	1.1%
New Infrastructure	0.00	0.00	0.0%	41.93	0.06	0.4%
**Total Capital Costs**	2531.59	2.88	16.1%	2003.25	2.78	21.2%
**RECURRENT COSTS**						
Personnel	10,130.33	11.55	64.2%	4910.71	6.80	51.9%
Supplies	2438.15	2.78	15.5%	2246.65	3.11	23.8%
Contracted services	637.35	0.73	4.0%	99.01	0.14	1.6%
Travel (gas mileage)	35.2	0.04	0.2%	152.28	0.21	1.1%
Utilities	8.36	0.01	0.1%	33.14	0.05	0.4%
**Total Recurrent Costs**	13,249.39	15.11	83.9%	7441.79	10.31	78.7
**TOTAL**	**15,780.98**	**17.99**	**100.0%**	**9445.04**	**13.08**	**100.0%**

**Table 3:** Cost-effectiveness results for MMC demand intervention strategy in Gauteng Province, South Africa 2015 (in USD)

	Standard of Care	Intervention	Difference (Intervention – Standard of Care)
**Number exposed to recruitment strategy**	9169	4839	
**Number of MMC Clients enrolled (mature men)**	877	722	
**% mature men enrolled**	9.56%	14.92%	5.36%
**Number of mature men recruited per 100 men exposed to recruitment strategy**	9.56	14.92	53.6
**Total Cost of MMC recruiting (USD)**	15,780.98	9445.04	
**Cost per 100 man exposed**	172.11	195.18	
**Cost per 100 man enrolled**	1799.00	1308.00	
**Incremental Cost Effectiveness Ratio (per 100 mature men enrolled)**	491.25		

### Cost-effectiveness and sensitivity analysis

Table 4 summarizes the cost-effectiveness results. Effectiveness is defined as the number of men enrolled in the standard care or intervention strategy. Approximately 5.4% more men aged 25–49 years (53.6 men per 100) were enrolled using the intervention strategy versus the standard care strategy. Results are presented for the 9169 men exposed to the standard care strategy and 4839 men exposed to the intervention strategy. For 100 men exposed, approximately 9 men were enrolled in the standard of care and approximately 14 in the intervention, with a difference of 6 men. The cost per every 100 men exposed to the intervention was $195. Results are also presented for the 877 men circumcised as a result of the standard care recruitment strategy versus the 722 men circumcised resulting from the intervention strategy. The cost per every 100 men circumcised as a product of the intervention was $1308. This was a reduction in the cost per every 100 men circumcised in the standard care scenario of $1799.

**Table 4:** Cost of intervention by separate intervention strategy in Gauteng Province, South Africa 2015 (USD)

Input	Structural modifications	VIP Facebook club	Imbizo meetings	Male friendly services	Community Demand generation material	Meetings	Total
**CAPITAL COSTS**							
Equipment	0	347.4	104.85	164.54	412.93	0	**1029.71**
Buildings	0	0	0	95.33	362.77	0	**458.10**
Training	0	0	365.79	0	0	0	**365.79**
Vehicles	0	0	0	0	107.72	0	**107.72**
New Infrastructure	41.93	0	0	0	0	0	**41.93**
**RECURRENT COSTS**							
Personnel	0	680.32	22.34	148.48	4049.84	9.73	**4910.71**
Supplies	0	0	38.86	31.02	2176.76	0	**2246.65**
Travel	0	0	0	0	152.28	0	**152.28**
Contracted Services	0	0	39.6	0	59.41	0	**99.01**
Utilities	0	3.87	1.17	12.28	15.66	0.16	**33.14**
**TOTAL**	**41.93**	**1031.59**	**572.61**	**451.66**	**7337.36**	**9.89**	**9445.04**
**PERCENT**	**0.4%**	**10.9%**	**6.1%**	**4.8%**	**77.6%**	**0.1%**	**100.0%**

Table 5 presents the number of HIV cases averted, total treatment costs averted, CBR and the net cost savings (net present value of the intervention). To obtain the total number of cases averted due to the intervention, the difference between the projected number of HIV cases that would have occurred without the intervention and the projected number of cases HIV cases that would occur among the participants receiving an MMC was calculated.

**Table 5:** Parameters for calculating averted HIV: base case values and sensitivity analysis ranges

Parameters (symbol)	HIV	Sources
**Disease Prevalence Rate** (*π*)		
For Participants	**0.21** (0.10–0.30)	[13]
For Partners	**0.29** (0.24–0.35)	[13]
**Probability of Transmission (***α***)**		
Female to male	**0.006** (0.003–0.01)	[19]
Male to female	**0.014**	[19]
**Condom Effectiveness (***σ***)**		
	**0.90** (0.87–0.95)	[19–21]
**Effectiveness of circumcision on HIV transmission** ***(v)***		
Male to female	**0.20** (0.10–0.30)	***Authors’ assumption***
Female to male	**0.60**	[1, 3, 22]
**Sexual Behavior parameters**		
Probability of condom use ***(f)***	**41.8%**	[14]
Average number of sex acts per partner ***(n)***	**27**	[14]
Average number of partners ***(m)***	**1.54**	[14, 23]

Among the 722 men aged 25–49 years enrolled in the intervention (and receiving MMC), an estimated total of 57 cases of HIV were averted. The annual cost of ART in South Africa in 2014/15 dollars was $269 [17]. Averted HIV costs were considered as life-time costs of ART-related costs from a provider’s perspective and comprised of the cost of antiretroviral drugs, CD4+ cell count and viral load monitoring, toxicity laboratory monitoring, and public clinic or private general practitioner visits. The costs of opportunistic infection and adverse event treatment were not included. Public-care and private care lifetime ART costs were $14,445 (USD 2014) and $10,067 (USD 2014) respectively [18].

Thus, total HIV treatment costs averted due to the intervention was $542,491 from a public-care model and $378,073 from a private-care model. Given the total cost of the intervention of $9445, the cost-benefit ratio was 57.44 for the public-care model, meaning for every dollar invested in the demand creation program, approximately $57 dollars in HIV treatment costs were saved if an individual was treated in the public system. Similarly, the cost-benefit ratio for the private-care model was 40.03, meaning for every dollar invested in the demand creation program, approximately $40 dollars in HIV treatment costs were saved if an individual sought care from the private system. The net benefit or cost savings of the intervention beyond the cost of the intervention was $533,046 and $368,628 for the public and private care model, respectively.

Table 6 presents values of the key parameters used in the Bernoulli model to estimate the cases of HIV averted, which were the results of the sensitivity analysis on the key epidemiologic/biological parameters and cost parameters. This includes HIV prevalence rates among the participants and their partners, probability of HIV transmission from male to female and from female to male, effectiveness of condoms in the prevention of HIV transmission, the effectiveness of circumcision on the transmission of HIV, and building and personnel costs are presented in Table 5. One-way sensitivity analysis on the epidemiological and cost parameters demonstrates that the results were robust, yielding favourable benefit-cost ratios. The parameters most sensitive to changes were the probability of HIV transmission rates and the participants and partners disease prevalence rates.

**Table 6:** Benefit-cost results for MMC demand intervention strategy in Gauteng Province, South Africa 2015 (USD)

HIV cases averted	HIV treatment cost to 60 years of age	Total costs averted	Total cost of intervention	Benefit cost ratio	Total cost savings
57	$26,923 – $9425	$153,489 – $537,278	$9445	7.41–25.94	$60,567.76 – $235,599.76

Cost parameters were also varied at +/− 20% for the sensitivity analysis as indicated in Table S1. Personnel costs varied due to the different positions that could execute the responsibilities. Building costs varied, as it was assumed that building costs for the Non-Governmental Organization (NGO) clinic was on the higher end. The benefit-cost ratio remained favourable with the variation in the cost parameters. Thus, it can be assume the results are robust and favourable with high or low disease prevalence rates and high or low-cost parameters.

## Discussion

This cost-effectiveness analysis of an “Exclusive Intervention Strategy” that implemented an innovative demand creation strategy for men aged 25–49 years for MMC in South Africa was more cost-effective than the standard care and resulted in lower costs per client enrolled. Costs for the intervention strategy were lower than the standard care. The cost savings could be further substantiated by the potential number of HIV cases averted. The benefit-cost ratio remained favourable with the +/− 20% variation in the cost parameters for the sensitivity analysis. Thus, it was assumed the results will prove favourable in regions with high or low disease prevalence rates and or at clinics with higher or lower costs.

Costing studies have shown that MMC is cost-effective, produces cost savings [5, 13], and should be included as part of HIV prevention packages. The results of this study showed that the cost per man aged 25–49 years enrolled for the intervention was lower when compared to the cost of demand creation activities from other studies [7, 14]. Probable reasons for our lower units costs were due to allocating demand creation costs to total number of males enrolled and not only to those circumcised, which was used in previous studies [7, 14].

Personnel, supplies, and equipment were the major cost drivers for base-case and intervention. The reduction in costs between the intervention and standard care could be due to the use of supplies targeted for this age group rather than increased personnel for demand creation. Other demand creation programs in South Africa have been within a continuum of activities for MMC, including circumcision. These studies, however, also had similar cost drivers of personnel costs [24] and supplies [25, 26].

Regarding cost categories for the intervention strategy, community demand, VIP Facebook and Imbizo meetings had the highest costs. The overall unit cost for demand creation compares favourably with unit costs for demand creation within South Africa. George et al. (2017) reported average cost per circumcision at $39, and $42 for demand strategies targeting adolescents [25]. These reductions in costs could be further improved by limiting the interventions to only those were effective (as described in Grund et al. [14], ,and by assuming that this intervention could be rolled out more widely and would reduce costs due to economies of scale (such as costs of production of pamphlets).

## Conclusions

### Strengths and limitations

This was one of the first studies in South Africa to assess the demand creation costs of reaching men 25–49 years of age for circumcision. This study also included novel intervention activities such as clinic structural modifications, use of Facebook, Imbizo discussions, and male-friendly services to recruit men for circumcision.

Despite these strengths, this costing study had limitations. The different methods of collecting time data may have influenced the cost results as interviews conducted retrospectively often involve recall bias which could over or underestimate the costs as compared to timesheets that are completed prospectively and are more accurate. Similarly, a formal time-motion study was not used to verify staff time spent performing various activities - instead observation and reviews of submitted time sheets were utilised to track activities. Labour costs are particularly high in South Africa compared to other sub-Saharan countries, and as the MMC demand creation teams are formally employed, rather than volunteers or lay-staff, the cost of the demand creation using outreach teams could have been overestimated compared to other countries and different labour structures. Building costs were estimated using rental rates for a similar facility as the rates for the actual facility were not available. It is possible that some participants who received supplies did not present at the clinic so those supplies would have been wasted. As a result, unit costs may be a slight overestimate as we did not account for this wastage. There are limitations with interpretation and generalizability of findings. In addition, it was assumed that the increased circumcisions are attributable to the intervention alone, however the study was not a cluster randomized trial, but rather an implementation science study. Despite these limitations, the study shows the importance of using low-cost methods to recruit men ages 25–49 years for MMC.

### Recommendations

The study illustrated that the use of effective methods targeting specific populations could lead to significant cost savings. Additionally, this study illustrated that some interventions, while seeming to initially increase costs, if effective, can result in significant cost savings. Based on the aforementioned research, it is recommended that using age-specific activities that increase word-of-mouth communication and should be explored further in demand creation strategies.

## Supplementary Information

**Additional file 1: Table S1.** Results of the Univariate and Multivariate Sensitivity Analysis [27–30].

## Data Availability

The data analyzed during the current study are available from the corresponding author on reasonable request.
