# Supplementary material for: An economic evaluation of an intervention to increase demand for medical male circumcision among men aged 25–49 years in South Africa
Source: BMC Health Serv Res. 2021 Oct 15;21:1097. doi: 10.1186/s12913-021-06793-7 (PMC8520207; doi:10.1186/s12913-021-06793-7)
Supplement: Supplementary file 1 — Additional file 1: Table S1. Results of the Univariate and Multivariate Sensitivity Analysis [27–30]. [file 12913_2021_6793_MOESM1_ESM.docx]

**Supplemental Material:**

**Estimation of Cases of HIV Averted and Costs Averted**

Following the approach of Pinkerton and colleagues (1,2,3), we used a mathematical model of sexual disease transmission to estimate the number of HIV cases averted by the intervention. We included primary infections (those occurring among the male participants) averted and secondary infections (those occurring among the sex partners of the male participants) averted. The cumulative probability for primary transmission was calculated for the intervention group (*P*). The following equation was used for our calculations.

$P = 1-\left[\left(1 - \pi\right) + \pi\left(1- fv\sigma\right) \alpha)^{n}\right] ^{m }$, (A.1)

where $\pi$ is the prevalence of the HIV among the sex partner(s), $\alpha$ is the per-act probability of HIV transmission per act of unprotected vaginal intercourse, *f* is the probability of condom use, *v* is the effectiveness of medical male circumcision in reducing the probability of HIV transmission, $\sigma$ is the effectiveness of condoms in reducing the probability of disease transmission, *m* is the number of sex partners, and *n* is the number of sex acts per partner. The number of secondary infections (S) among an infected participant’s partners (m) was calculated as:

$S = m \left(1 - \pi\right) \left\{ 1 - \left(1 - \left(1 - fv\sigma\right)\alpha\right)^{n}\right\}$. (A.2)

We converted the 3-month probabilities into 1-year probabilities by using the following:

$P^{k} = \left[1 - \left(1 - P\right)^{4}\right]$ and $S^{k} = \left[1 - \left(1 - S\right)^{4}\right]$ (A.3)

The expected number of infections among the men participating in the intervention and their partners were calculated as$N\left[ (1-\theta)P + \theta S\right]$, where N is the number of group participants and $\theta$ is the prevalence of HIV among the participants. Thus, there are N (1-$\theta$) participants at risk of acquiring HIV, and N$\theta$ participants at risk of transmitting HIV. The estimated number of infections averted for the intervention was the sum of the primary and secondary infections.

We made several simplifying assumptions. We only considered outcomes averted due to vaginal intercourse since the majority of HIV risk-related behavior in this population occurred in the context of heterosexual relationships and the percentage of students participating in anal sex was less than 3% (4). We assumed that up to 30% of the partners were 5 years younger than the men, up to 67% were in the same age group as the participants, up to 2.7% % were 5 years older than the participants. These percentages were derived from self-reports of the mature men participating in the Imbizo intervention (4). We assumed effects of the intervention lasted for the year. We assumed condoms were the only contraceptives used.

Behavioral data were also obtained from the Imbizo intervention (**4**). Following the approach of Pinkerton et al, we calculated the annual numbers of acts of vaginal intercourse by multiplying the 3-month mean number of acts by four, and we conservatively assumed that the annual number of sex partners was the same as the 3-month mean number. The value of HIV prevalence among the participants and their partners was 21% and 29% respectively, based on calculations from the South African National HIV Prevalence, Incidence and Behaviour Survey. We used published estimates on condom effectiveness. All parameter values for equations A.1, A.2, and A.3 are presented in Table S.1.

**Table S1: Results of the Univariate and Multivariate Sensitivity Analysis**

|  |  | **Cases**  **Averted** | **Summary Measures** | | | |
| --- | --- | --- | --- | --- | --- | --- |
| **Parameters (symbol)** | **Value** | **HIV** | **Public**  **Benefit**  **Cost**  **Ratio** | **Public**  **Net**  **Present**  **Value** | **Private**  **Benefit**  **Cost**  **Ratio** | **Private**  **Net**  **Present**  **Value** |
| **Univariate Sensitivity Analysis** | | | | | | |
| **Disease Prevalence Rate** ($\pi$) - Partners |  |  |  |  |  |  |
| *Partners* | 0.24 – 0.35 | 27 - 48 | **42.35 – 73.61** | **$384,234 - $684,806** | **29.39 – 51.30** | **$266.698 - $473,158** |
| *Participant* | 0.10 – 0.30 | 37 - 38 | **51.65-64.43** | **$531,914 - $534,303** | **35.97 – 45.13** | **$367,512 - $369,735** |
|  |  |  |  |  |  |  |
| **Probability of Transmission ($\alpha$)** |  |  |  |  |  |  |
| *Female to male* | 0.0011 – 0.0125 | 0.00 – 133.18* | **0.00 – 203.46** | **$0 - $1,891,846** | **0.00 – 142.21** | **$0 - $1,330,172** |
| *Male to female* | 0.0008 – 0.002 | 36 – 38 | **56.15 – 58.68** | **$131,896 - $574,341** | **39.14 – 40.90** | **$360,998 - $376,998** |
|  |  |  |  |  |  |  |
| ***Condom Effectiveness***  ***(*)** | 0.87 – 0.95 | 35 - 40 | **54.35 – 61.63** | **$504,092 - $572,601** | **37.87 – 42.98** | **$349,113 - $396,520** |
|  |  |  |  |  |  |  |
| **Effectiveness of circumcision on HIV transmission male to female *(v)*** | 0.01 –  0.03 | 32 - 42 | **49.98 –**  **64.97** | **$462,652 –**  **$604,205** | **34.83 – 45.27** | **$319,543 - $418,246** |
|  |  |  |  |  |  |  |
| **Costs** |  |  |  |  |  |  |
| **Personnel Costs** | +/- 20% | n/a |  |  | **8.89 – 30.13** | **$74,031.80 - $282,723.80** |
| **Building Costs** | +/- 20% | n/a | **56.88 – 57.99** | **$532,955 – 533,138** | **39.64 – 40.42** | **$368,536 - $368,628** |
|  |  |  |  |  |  |  |
| **Multivariate Analysis** | | | | | | |
| **All parameters except female to male HIV transmission^1^** | All parameters varied per above | 22 - 53 | **31.76 – 91.04** | **$311,332 - $766,482** | **27.74 – 59.77** | **$205,159 - $534,839** |
| ***Lower bound was presented as 0. Sensitivity analysis yielded a lower bound of a negative number.**  **^1^The female to male HIV transmission rate was not included due to negative numbers in the lower bound found in one-way sensitivity analysis.** | | | | | | |

***REFERENCES FOR SUPPLEMENTAL MATERIAL***

1. Gopalappa, C., Huang, Y.A., Gift, T.L, Owusu-Edusei, K., Taylor, M., & Gales, V., (2013). Cost-Effectiveness of Screening Men in Maricopa County Jails for Chlamydia and Gonorrhea to Avert Infections in Women. *Sex Transm Dis*. 40(10): 776–783. doi:10.1097/OLQ.0000000000000023.
2. Pinkerton, S.D.; Abramson, P.R.,(1998). The Bernoulli-process model of HIV transmission: Applications and implications. In: Holtgrave, R., editor. Handbook of Economic Evaluation of HIV Prevention Programs. New York: Plenum Press; p. 13-32.

1. Pinkerton, S.D., (2012). HIV Transmission Rate Modeling: A Primer, Review, and Extension. AIDS Behavior 16 (4): 791 – 796. doi:10.1007/s10461-011-0042-8.
2. Grund JM, Chetty-Makkan CM, Ginindza S, Munyai R, Kisbey-Green H, Maraisane M, et al. Effectiveness of an “Exclusive Intervention Strategy” to increase medical male circumcision uptake among men aged 25–49 years in South Africa. BMC Public Health [Internet]. 2018;18(1):868. Available from: https://bmcpublichealth.biomedcentral.com/articles/10.1186/s12889-018-5729-6
